# Recent Progresses in the Multimodality Imaging Assessment of Myocardial Fibrosis

**DOI:** 10.31083/j.rcm2501005

**Published:** 2024-01-08

**Authors:** Han Zhu, Kewei Xie, Yingying Qian, Zhiguo Zou, Meng Jiang, Jun Pu

**Affiliations:** ^1^Department of Cardiology, Renji Hospital, Shanghai Jiao Tong University School of Medicine, 200127 Shanghai, China; ^2^Department of Nephrology, Renji Hospital, Shanghai Jiao Tong University School of Medicine, 200127 Shanghai, China; ^3^Department of Nephrology, Affiliated Hangzhou First People's Hospital, Zhejiang University School of Medicine, 310011 Hangzhou, Zhejiang, China; ^4^State Key Laboratory of Systems Medicine for Cancer, Shanghai Cancer Institute, Renji Hospital, Shanghai Jiao Tong University School of Medicine, 200127 Shanghai, China

**Keywords:** myocardial fibrosis, multimodality imaging assessment, nuclear medicine, echocardiography, cardiac magnetic resonance

## Abstract

Myocardial fibrosis, a common pathophysiological consequence of various cardiovascular diseases, is characterized by fibroblast activation and excessive deposition of extracellular matrix (ECM) collagen. Accumulating evidence indicates that myocardial fibrosis contributes to ventricular stiffness, systolic and diastolic dysfunction, and ultimately leads to the development of heart failure (HF). Early detection and targeted treatment of myocardial fibrosis is critical to reverse ventricular remodeling and improve clinical outcomes in patients with cardiovascular diseases. However, despite considerable progresses made in understanding molecular mechanisms of myocardial fibrosis, non-invasive imaging to assess myocardial fibrosis and guide clinical treatment is still not widely available, limiting the development of innovative treatment strategies. This review summarizes recent progresses of imaging modalities for detecting myocardial fibrosis, with a focus on nuclear medicine, echocardiography and cardiac magnetic resonance (CMR).

## 1. Introduction

**Fig. 1.**
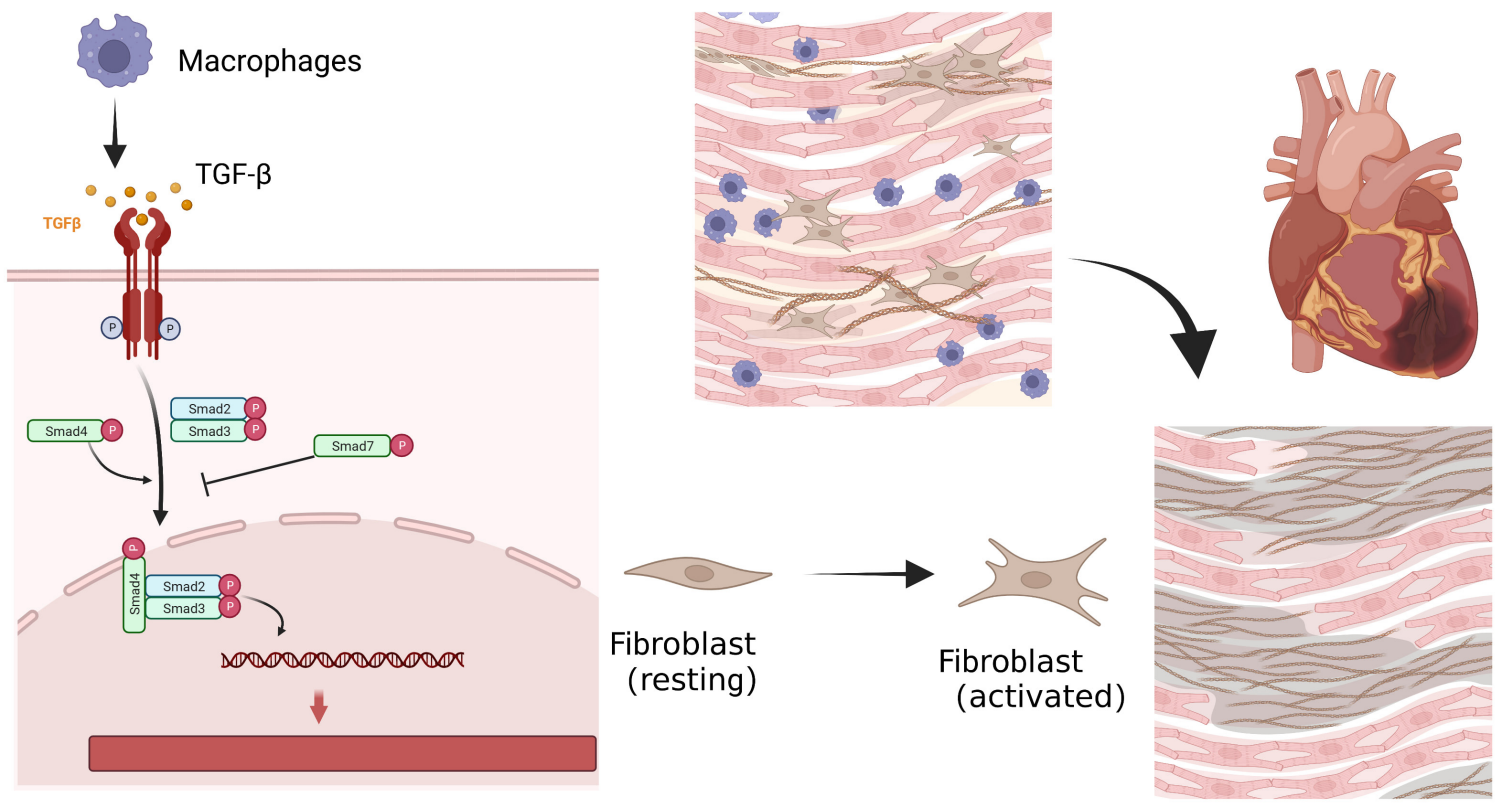
**Cellular process of myocardial fibrosis after myocardial infarction**. Repairing macrophages are recruited to engulf apoptotic neutrophils, and release inhibitory transmitters such as transforming growth factor-β and other potent inflammatory inhibitors. These factors can induce the activation of resting fibroblasts with higher expression of fibroblast activation protein (FAP), and trigger the profibrotic process. While cardiac scar tissue maintains the structural integrity and pressure-generating capacity, persistent myocardial fibrosis leads to adverse changes in the structure and compliance of the ventricles, resulting in the progression of heart failure (HF).

Myocardial fibrosis, defined as an excessive accumulation of extracellular matrix (ECM) proteins, results in pathological ventricular remodeling and, eventually leads to heart failure (HF) [1]. Myocardial fibrosis can be divided into several subtypes including: replacement fibrosis, reactive interstitial fibrosis, endomyocardial fibrosis [2] and infiltrative interstitial fibrosis. Reactive interstitial fibrosis is an adaptive, non-specific response distinguished by a scattered microscopic distribution in the myocardium, occasionally accompanied by local peripheral distribution of blood vessels [3], with sustained activation of pro-fibrotic growth factors including transforming growth factor-β (TGF-β) (Fig. 1), fibroblast growth factor-2 and connective tissue growth factor [4]. Such interstitial form of fibrosis is typically secondary to long-term pressure and volume overload, resulting in hyperactive renin-angiotensin-aldosterone system and adrenergic system, as presented in valvular heart disease [5], chronic hypertension [6], and cardiomyopathies such as hypertrophic cardiomyopathy [7], dilated cardiomyopathy [8] and diabetic cardiomyopathy [7], but also in distal non-infarcted myocardium following myocardial infarction [9]. Early mild replacement interstitial fibrosis is reversible with specific treatment [10]. Infiltrative myocardial fibrosis is caused by excessive storage of misfolded, insoluble, aggregated proteins (amyloidosis) [11] or globotriaosylceramide (Fabry disease) [12] in the extracellular matrix. Replacement myocardial fibrosis often occurs after acute myocardial infarction (AMI), where necrotic myocardial cells are replaced by collagen fibers, forming fibrous cardiac scar, which ensures the integrity of the heart from rupture in the early stages of myocardial infarction [13]. On the other hand, if left untreated and overburdened post-AMI, the fibrotic tissue can spread to the non-infarcted myocardium, resulting in decreased tissue compliance and cardiac dysfunction [14]. In addition, the excessive deposition of ECM damages the mechanical-electrical coupling of myocytes, impairing myocardial contractility and raising the incidence of malignant arrhythmias and sudden death [15]. In addition, epidemiological studies and clinical trials have shown that myocardial fibrosis is an independent risk factor of adverse cardiac events such as AMI, HF, arrhythmia and cardiovascular death [7].

Myocardial fibrosis can be detected by a variety of methods in clinical practice. Traditionally, endomyocardial biopsy is the gold standard for determining myocardial fibrosis, despite its invasive and inconvenient properties. In addition, the diagnosis of myocardial fibrosis by endomyocardial biopsy can be challenging due to its low diagnostic yield, especially for diffuse myocardial fibrosis [16, 17]. In the past, imaging examination such as electrocardiogram and echocardiography were applied to observe cardiac electrical conduction, cardiac structure, and function. In recent years, novel imaging techniques have provided more evidence for determining the characteristics of myocardial tissue, such as single-photon emission computed tomography (SPECT) and cardiac magnetic resonance (CMR). Different imaging tests have their unique features. Since multimodality imaging plays an important role in the initial assessment and diagnosis of myocardial fibrosis, here we discuss current available noninvasive imaging techniques and their values in guiding clinical treatment and improving patient outcomes.

## 2. Echocardiography

Echocardiography, based on the principle of ultrasonic ranging, is a preferred non-invasive technique to examine the anatomical structure and function of the heart and great vessels [18]. Echocardiography has outstanding advantages such as convenience, rapidity, and non-invasively bedside use. Fibrosis can be hinted when structural and functional changes such as abnormal myocardial thickening, and systolic or diastolic dysfunction are observed [19]. The strategy of integrated backscatter analysis (IB) in standard 2-dimensional (2D) ultrasound images is the first attempt for noninvasive evaluation of myocardial fibrosis after infarction using echocardiography [20]. It measures two parameters of ultrasonic tissue characterization: the amplitude of the cardiac cycle-dependent variation of the backscatter integral signal (cdv-IB) and the mean value of IB [21]. IB signal calibrated by the backscatter power from the pericardium. Moreover, in patients with dilated or hypertrophic cardiomyopathy, m-IB during a cardiac cycle was reported to correlate with the severity of myocardial fibrosis [22]. The intensity of septal IB signal increases in patients with hypertrophic cardiomyopathy (HCM). As a marker of interstitial fibrosis, it is associated with a progressive increase in Doppler parameters related to ventricular stiffness such as pulmonary venous backward velocities and mitral peak velocity at atrial contraction [23]. Losi and colleagues [23] further showed that in HCM patients, the occurrence of ventricular tachyarrhythmias was significantly associated with higher IB signal rather than septal thickness. In addition, echocardiographic measurements based on backscatter techniques include signal intensity coefficient (SIC), which utilizes the greyscale signal intensity values generated at the myocardium-pericardium interface resulting from interactions between the ultrasound signal and myocardial tissue [24]. SIC produces measurable differences between diseased and healthy myocardium. In populations carrying genetic variants associated with HCM, SIC values significantly correlate with left ventricular (LV) hypertrophy [24]. However, this observation is not applicable in patients with coronary artery disease. Higher calibrated integrated backscatter (cIB) was not confirmed as a marker of increased myocardial fibrosis, but was associated with higher soluble vascular endothelial growth factor receptor-1 (sVEGFR-1) and soluble receptor for advanced glycation end products (RAGE) plasma levels. Meanwhile, correlation between cIB and myocardial fibrosis has not been proven by histological examination and CMR evaluation [25, 26].

### 2.1 Pulse-Cancellation Echocardiography

In 2016, Dr. Gaibazzi and colleagues [19, 27] attempted to identify myocardial scar or fibrotic areas using “echocardiographic scar” (eScar). This technique combined 2D ultrasound imaging with multipulse modulation and inversion to achieve a higher spatial and temporal resolution than 3D imaging. Compared with standard harmonic imaging, eScar is designed to distinguish scars from normal myocardium. Using the CMR-late gadolinium enhancement (LGE) technique as reference, eScar has been proven to be able to identify the presence and location of cardiac scars in patients with ST-elevation myocardial infarction (STEMI) [19]. While, sensitivity in apical myocardial segments, quality of image, and gain dependence are still noteworthy problems for eScar echocardiography [19]. Nevertheless, eScar has been applied in the prediction of appropriate implantable cardioverter-defibrillator (ICD) shocks in patients after myocardial infarction [28]. Intriguingly, eScar also shows the ability to assess subclinical myocardial involvement and predict disease activity in patients with systemic lupus erythematosus (SLE), an autoimmune disease involving multiple systems throughout the body [29]. In this pilot study, eScar identified myocardial scars at the inferoseptal myocardial segments in 19% of SLE patients while in none of the controls. Therefore, as a rapid and inexpensive technique, eScar can be routinely applied in routine clinical practice for cardiac monitoring in patients with multi-organic diseases such as SLE [29].

### 2.2 Two-Dimensional and Three-Dimensional Speckle Tracking Echocardiography

Myocardial strain including global longitudinal strain (GLS), global circumferential strain (GCS), global radial strain (GRS) and tangential strain (TS) from the scatter-tracking technique have been applied to assess fibrotic myocardium [30]. In general, GLS are recommended as the most sensitive myocardial deformation parameter, which reflects impaired subendocardial fibres [31]. Using CMR as a reference, echographic GLS is significantly related to the estimated degree of fibrosis in patients with HCM [31], and Anderson-Fabry disease [32, 33], and heart transplant recipients [34]. However, similar correlations were not observed between GCS, GRS and myocardial fibrosis.

Patients with advanced heart failure prominent present with right ventricular (RV) enlargement, increased myocardial fibrosis and systolic dysfunction. Myocardial deformation of the RV free wall is one of the most accurate functional indicators and is associated with RV myocardial fibrosis and functional capacity [35]. Longitudinal strain from speckle tracking echocardiography has been proven useful in assessing the severity of right ventricular fibrosis [36].

Novel parameters including mechanical dispersion and myocardial work are able to offer additional possibilities for the evaluation of myocardial fibrosis. Both mechanical dispersion (the standard deviation of the time to peak negative strain in LV segments) and myocardial work (reflects the stroke work of the pressure-strain circuit by combining LV deformation and afterload information) have been reported in pilot studies as stronger predictors of LV myocardial fibrosis compared to GLS [37, 38].

## 3. Cardiac Magnetic Resonance (CMR)

CMR has become the preferred imaging modality for evaluating myocardial fibrosis due to its ability in soft tissue characterization. T1-weighted images for scar and T2-weighted images for edema visualization are essential sequences to characterize soft tissue [39]. CMR imaging-derived parameters, particularly by LGE and T1 mapping sequences, are widely used to identify fibrotic myocardium. LGE can depict local replacement myocardial fibrosis as seen in large focal post-infarct scars, while T1 mapping has the potential in detecting and quantifying diffuse myocardial fibrosis, since it evaluates the T1 relaxation time of myocardial tissue [40].

### 3.1 Late Gadolinium Enhancement

LGE is a clinically useful non-invasive CMR sequence for the detection of focal cardiac fibrosis. The reduced density of capillaries in the fibrotic myocardial tissue leads to a higher concentration of the contrast agent retained in the fibrotic region [41]. Graphically, fibrotic tissue was significantly enhanced on LGE images compared to normal myocardial tissue [42]. In patients with myocardial infarction, a delayed contrast enhancement by magnetic resonance imaging (MRI) was recommended to distinguish viable from non-viable myocardium throughout the infarct healing process [43]. Furthermore, a significant correlation was found between LGE and collagen deposition in the myocardial tissue, which is an indirect indication of fibrosis severity as measured by extracellular matrix volume [44, 45].

The use of LGE is rapidly expanding to assess myocardial fibrosis in cardiomyopathies [46]. Several patterns of LGE that are distinct from ischemic cardiomyopathy have been identified. However, these patterns are not specific enough to be used as diagnostic criteria [47]. Around one third of patients with dilated cardiomyopathy presented non-ischemic LGE pattern (mid-lateral or subepicardial), which is also a predictor of adverse cardiovascular events, including heart failure, ventricular arrhythmias, sudden cardiac death (SCD) and all-cause mortality [48]. Patchy fibrosis in the mid-ventricular layer is the typical pattern characterized by LGE in patients with HCM [49]. Epidemiological study has shown that HCM-related myocardial fibrosis is closely related to arrhythmia, and is remarkably associated with subsequent SCD after adjusting for other risk factors [49]. Moreover, a recent meta-analysis demonstrated LGE as the single best imaging marker to predict adverse outcomes in HCM patients [50].

In addition to risk prediction, the severity of myocardial fibrosis assessed by LGE CMR can be used to guide clinical treatment, such as optimization of the timing of ICD implantation [51]. In addition, LGE CMR-based assessment of myocardial fibrosis plays an important prognostic role in aortic stenosis, Eisenmenger’s syndrome, hypertension and diabetes mellitus [52, 53].

Despite increasing applications of LGE CMR, the setting of intensity threshold for cardiac fibrosis by LGE imaging is still not clear in clinical practice [44]. Scarred myocardium is defined as higher signal intensity than normal myocardium in LGE, and official guidelines advocate a threshold of 2-standard deviation (SD) [54]. However, other techniques also can be applied, including the 3, 4, 5, or 6 SD method, manual quantification (mapping the region of interest around the scar), and the full width at half maximum (FWHM) technique that uses half of the maximal signal within the scar as a threshold. LGE volume varied substantially depending on the quantification method used. The 2-SD technique produced a 2-fold higher LGE volume than the FWHM, 6-SD and manual techniques, while the FWHM technique displayed the best reproducibility [55].

Additionally, since the LGE interpretation is based on the difference of contrast agent distribution among tissues, the application in diffuse myocardial fibrosis detection was not feasible. Also, the increased extracellular matrix due to inflammation and edema may lead to interpretation errors in the assessment of fibrotic myocardium [44].

### 3.2 T1 Mapping

T1 mapping technique has the advantage in detecting diffuse myocardial fibrosis resulting from valvular disease or various cardiomyopathies. In contrast to LGE, T1 mapping does not depend on the contrast between normal and scarred myocardium. It provides a quantitative assessment of the tissue characterization based on a fully quantitative pixel analysis. In combination with hematocrit, these data allowed the quantification of extracellular volume (ECV) to evaluate myocardial fibrosis. ECV fits well with the histological extracellular space. Both T1 mapping and ECV has shown high reproducibility in detecting and quantifying histological collagen volume fractions [56].

Alternative fibrosis often occurs after myocardial infarction, and T1 mapping sequence can dichotomously identify infarct areas as a potential tool for measuring infarct size [57], which showed good agreement between native T1 mapping and LGE imaging modality [58]. In patients with severe aortic valve disease, diffuse myocardial fibrosis assessed by anterior septal-basal ECV correlates with histological myocardial fibrosis. Prolonged T1 value and elevated ECV can also be detected in dilated cardiomyopathy suggesting the presence of myocardial fibrosis occurrence [59]. T1 and ECV in detecting fibrosis have also been studied in hypertrophic cardiomyopathy. Even in the absence local LGE and hemodynamic obstruction, prolonged myocardial T1 and increased ECV suggest diffuse myocardial fibrosis in patients with HCM, which is also associated with left ventricular hypertrophy [60]. Native T1 and ECV quantification show high diagnostic performance for cardiac amyloidosis and can be used as non-invasive markers to assess disease severity and prognosis [61, 62]. The location and pattern of fibrosis favor the separation between healthy and fibrotic myocardium [63] and can distinguish hypertrophic cardiomyopathy from other hypertrophic heart diseases such as hypertensive heart disease [64].

Although CMR is currently the recommended imaging modality for clinical detection of myocardial fibrosis, patients with metal implants or pacemakers are prohibited to undergo CMR examination. Claustrophobic patients who have difficulty in overcoming psychological barriers to accept long time onboard examinations, and patients with congestive heart failure are usually not able to tolerate prolonged lying down. Moreover, normal range of T1 threshold is sensitive to the physical properties of contrast agent, acquisition time, and renal function and hematocrit of patients [44].

## 4. Computed Tomography (CT)

Recently, animal and clinical studies have demonstrated the feasibility of contrast enhanced CT in detecting fibrosis by CT delayed enhancement (CT-DE). The principle of CT-DE is similar to that of CMR LGE [65]. CT-DE allows quantitative assessment of ECV to evaluate fibrosis. CT-based ECV quantification is effective in assessing myocardial fibrosis, showing a strong correlation with CMR findings. CT-ECV also displayed high diagnostic accuracy in distinguishing LGE-positive from LGE-negative segments [65]. Furthermore, previous study indicated that CT was able to assess myocardial fibrosis in cases where CMR is not available, which still requires verification by further large-scale studies [66]. However, despite excellent specificity, the clinical use of CT-DE is limited by its low sensitivity. The study by Bettencourt *et al*. [65] showed a sensitivity of 53% and a specificity of 98% in 105 patients with suspected coronary artery disease.

Although higher volume of iodinated contrast agents and lower energies improve spatial resolution, the contrast difference between normal and infarcted myocardium detected by CT-DE is suboptimal compared to CMR [67]. To circumvent this limitation, dual-energy CT improves the characterization of tissue composition and image quality by using an X-ray source that emits 2 different spectra or by employing a 2-layer detector to achieve continuous acquisition of CT in different photon spectra [68].

## 5. Nuclear Medicine

Nuclear medicine a well-established advanced imaging modality for the diagnosis, and evaluation of cardiovascular disease. The combination of radionuclide imaging with biologically targeted molecules provides unique insight into disease mechanisms at the molecular level, which allows an early detection of damaged myocardium before pathological changes occur.

Myocardial fibrosis is recognized as excessive deposition of collagen. The collagen-targeted contrast agent is the first targeted probe for the detection of myocardial fibrosis after a heart attack. ^99m^Tc-streptavidin-coupled-collagelin and ^99m^Tc-CBP1495 are two collagen-targeting peptide tracers that have relatively high affinity for collagen. Significantly increased uptake of these tracers was observed in fibrotic tissues of rat models [69]. However, collagen-targeted peptides can only show the late products of myocardial fibrosis, and thus they are not sensitive for fibrosis detection at earlier stages of the disease. It is also not possible to determine whether myocardial fibrosis is ongoing. Velikyan *et al*. [70] recently reported a ^68^Ga-labelled collagelin analogue, which showed promises for the detection of early active fibrosis by binding to monomeric collagen before the collagen fibres mature. However, there is still plenty of uncertainty for clinical application.

Molecular targets of activated fibroblasts at early disease stages are predictive of the extent and severity of cardiac fibrosis. In a rat model of myocardial fibrosis, angiotensin II (Ang II) was highly expressed in activated macrophages and myofibroblasts. Through acting on Ang II type 1 receptors (At1R), Ang II induced the expression of TGF-β, which is the growth factor most closely associated with the development of tissue fibrosis [71]. Positron emission tomography (PET) experiments using ^11^C-KR31173 in a porcine myocardial infarction model suggested that the radioactive probe detection of At1R is feasible. The application in human were safe, and showed detectable retention of specific myocardial markers, but at lower levels than that in pigs [72]. Cy5.5-Arg-Gly-Asp (RGD) imaging peptide, a targeting marker for myofibroblasts, can also display interstitial changes in myocardial remodeling and assess fibrosis in response to anti-angiotensin therapy [73]. In the early post-myocardial infarction period, the range of tracer uptake measured by 99Tc-RGD imaging after 3 weeks is comparable to that of CMR imaging. The scar size shown by 99Tc-RGD imaging predicts the eventual scar formation after myocardial infarction.

Fibroblast activation protein (FAP) is expressed at high levels in activated fibroblasts and shows low expression in most normal organs [74]. Radioactively labeled fibroblast activation protein inhibitor (FAPI) is developed to detect activated fibroblasts and initially shows great promise in the diagnosis and treatment of cancer patients. The application of FAPI in cardiovascular disease began with the incidental observation of FAPI in cancer patients by PET. A correlation between tracer uptake and reduced ejection fraction has been observed by FAPI-PET imaging in patients with metastatic cancer [75]. FAPI binds to FAP and accumulates strongly in tissue with high fibroblast activation, showing a high bright signal compared to normal myocardium, with a low background signal. By exploiting the molecular characteristics of myocardial fibrosis, where fibroblasts are highly activated to produce collagen fibers, FAPI can be used as a specific target for the management and treatment of cardiovascular disease. More recently, it has been utilized in murine models and in humans for the assessment of myocardial fibrosis following myocardial infarction (MI) [76, 77, 78]. Serial imaging with ^68^Ga-FAPI in a MI model established by coronary artery ligation showed intense radiotracer uptake around the infarcted area, and the uptake peaked at day 6 [76]. In the study by Zhang *et al*. [79] aseline uptake volume (UV) was a powerful predictor of LV remodeling at 1 year after STEMI in 26 patients with ST-segment elevation myocardial infarction (STEMI) who underwent Ga-DOTA-FAPI-04 PET (OR = 1.048, *p* = 0.011). Lyu *et al*. [80] found that FAPI imaging was able to detect myocardial fibrosis in diabetic, obese and elderly patients, providing additional evidence for early intervention and clinical decision-making in the management of patients at elevated risk of CVD.

In addition, ^68^Ga-FAPI PET has been used in a rat model of HF to visualize myocardial fibrosis and monitor HF progression [79]. A study by Guokun Wang *et al*. [81] showed that fibroblast activation in the heart and liver after pressure overload could be monitored using ^68^Ga-FAPI-04 PET/CT and that this non-invasive technique was a better predictor of subsequent worsening of heart failure. FAP activity is heterogeneously increased in the myocardium of patients with hypertrophic cardiomyopathy, and their PET-measured FAPI uptake is a potential predictor for 5-year risk of sudden death from cardiovascular causes [82].

However, the cost of test, the worries about radiation, and the poor understanding of nuclear medicine have limited its use clinically. In the future, if these obstacles can be overcome, it will open a new era of targeted treatment and management of patients with myocardial fibrosis.

## 6. Conclusion and Future Perspective 

**Fig. 2.**
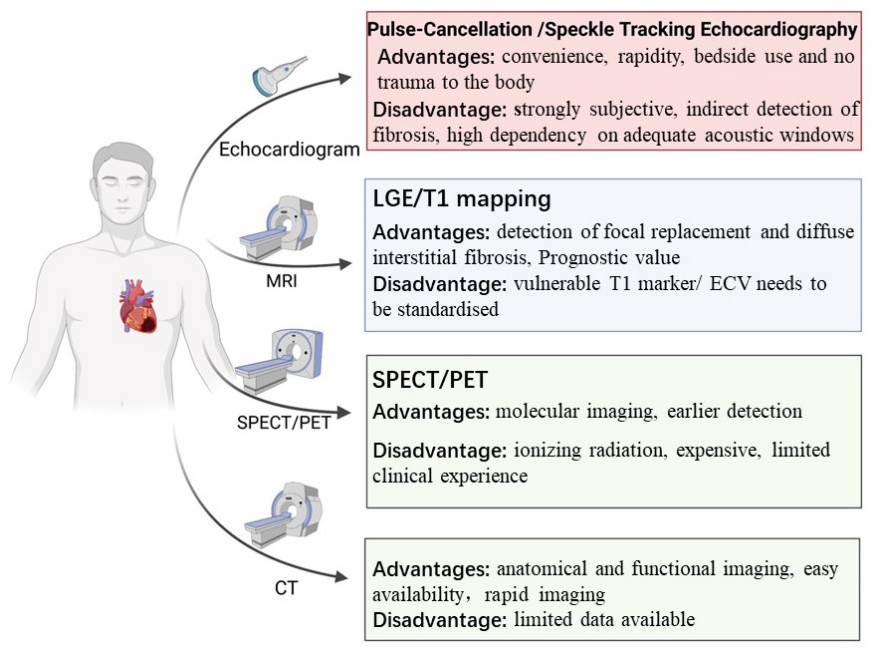
**Multimodality imaging assessment of myocardial fibrosis**. The multimodality imaging approaches that are able to assess myocardial fibrosis in clinical practice. LGE, CMR-late gadolinium enhancement; PET, positron emission tomography; MRI, magnetic resonance imaging; ECV, extracellular volume; CT, computed tomography; SPECT, single-photon emission computed tomography.

Early detection and targeted treatment of myocardial fibrosis is essential to improve clinical outcomes in patients with cardiovascular diseases. Multimodality non-invasive imaging approaches can directly or indirectly evaluate the presence and severity of cardiac fibrosis, with advantages and disadvantages of each technique summarized in Fig. 2. In summary, CMR is the gold standard for noninvasive detection and quantification of myocardial fibrosis in clinical practice, whereas other techniques show promises as valuable alternatives. Molecular imaging is developing rapidly and has been a promising technique not only for studying pathological mechanisms, but also for investigating the efficacy of individualized therapeutic regimens to meet the growing need for precision medicine. All the progresses made in the development of novel radiopharmaceuticals targeting specific cardiovascular molecules indicated that the revolution in personalized medicine has only just begun.
